# Dual Diagnosis, Double Trouble

**DOI:** 10.1192/j.eurpsy.2022.1197

**Published:** 2022-09-01

**Authors:** J. Lopes, R. Freitas

**Affiliations:** Hospital Espirito Santo, Departamento De Psiquiatria E Saude Mental, Evora, Portugal

**Keywords:** substance abuse, comorbidity, dual diagnosis

## Abstract

**Introduction:**

Many individuals with severe mental illness (SMI) have substance use disorder comorbidity. Dual diagnosis makes the approach and management of these patients even more challenging since the lack of improvement in either pathologies can lead to a deterioration of both.

**Objectives:**

To illustrate, through the presentation of two cases, the clinical challenges in managing a patient with dual diagnosis

**Methods:**

Clinical case presentation through retrospective review of clinical notes and non-systematic literature review on this topic

**Results:**

We present the clinical cases of two women diagnosed with Bipolar Disorder and (poly)Substance Use Disorder since adolescence, who have a history of multiple hospitalizations due to mostly maniform symptoms. The complexity of case management is evident, both at the pharmacological level and in psychosocial intervention. This is aggravated by the difficulty in maintaining adherence to the therapeutic project and frequent relapses.

**Conclusions:**

Current evidence points to the beneficial effect of a combined pharmacological and psychosocial approach, which must be comprehensive, individualized and require differentiation at various levels that are difficult to achieve and make the treatment of these situations an even greater challenge.

Using illustrative examples, this review draws attention to the practical difficulties in managing situations where substance use is associated with SMI.

**Disclosure:**

No significant relationships.

